# Bibliometric insights into astrocytic roles in depression and treatment

**DOI:** 10.3389/fncel.2024.1521398

**Published:** 2025-01-15

**Authors:** Linsun Lin, Ziyi Guo, Zhuoyu Ren, Yanchen Feng, Peigang Fang, Tao Wang, Min Chen

**Affiliations:** ^1^Faculty of Chinese Medicine and State Key Laboratory of Quality Research in Chinese Medicines, Macau University of Science and Technology, Macau, Macao SAR, China; ^2^Huizhou Health Sciences Polytechnic, Huizhou, China; ^3^National Engineering Laboratory for Internet Medical Systems and Applications, First Affiliated Hospital of Zhengzhou University, Zhengzhou, China; ^4^Department of Anesthesiology, Pain and Perioperative Medicine, The First Affiliated Hospital of Zhengzhou University, Zhengzhou, China; ^5^The First Clinical Medical School, Henan University of Chinese Medicine, Zhengzhou, China; ^6^Encephalopathy Center, The Second Affiliation Hospital of Anhui University of Chinese Medicine, Hefei, China; ^7^Guangdong Medical University, Zhanjiang, China

**Keywords:** depression, astrocyte, bibliometric, CiteSpace, VOSviewer

## Abstract

**Objective:**

Depression is a mental disorder that significantly impairs both physical and mental health. Recent studies have shown that reactive astrogliosis have gained significant attention for their involvement in the pathophysiology of depression. However, there is no bibliometric analysis in this research field. This study aims to provide a comprehensive overview of the knowledge structure and research hotspots regarding the role of astrocytes in the mechanisms and treatment of depression through bibliometric analysis. The scope of the literature review encompasses both basic and clinical research.

**Methods:**

Publications related to astrocytes in depression and treatment from 2014 to 2023 were searched in the Web of Science Core Collection (WoSCC) database. VOSviewer, CiteSpace, and the R package “bibliometrix” were used to conduct this bibliometric analysis.

**Results:**

From 2014 to 2023, a total of 1,502 documents from 78 countries on astrocytes in depression and treatment were analyzed from 169 journals, with the most co-cited journals being the Journal of Neuroscience and PNAS. China Medical University was the most productive institution. The analysis identified key authors like Verkhratsky Alexei and Baoman Li, and major co-cited references by Rajkowska and Liddelow. Keywords such as “synaptic plasticity,” “astrocytes,” and “neuroinflammation” revealed research trends focusing on molecular mechanisms, gut microbiota, and inflammation.

**Conclusion:**

This is the first bibliometric study to comprehensively summarize the research trends and advancements regarding astrocytes in depression and its treatment. Through this bibliometric analysis, we aim to enhance the understanding of the significance of astrocytes in depression research and provide new perspectives and insights for future investigations. We hope that this study will facilitate a broader integration of basic and clinical research, offering novel approaches for the treatment of depression.

## 1 Introduction

Depression is a widespread mental health disorder characterized by persistent sadness, loss of interest or pleasure in daily activities, and a range of emotional and physical problems (Smith, 2014). It is the leading cause of disability worldwide, significantly impacting the quality of life and daily functioning of those affected. The WHO reports 322 million people have depression and 260 million have anxiety, with rising prevalence globally (Friedrich, 2017). In addition, depression disorders are one of the leading causes of suicide and represent a significant public health problem (Orsolini et al., 2020). The pathophysiology of depression is complex and multifactorial, involving genetic, biochemical, environmental, and psychological factors. Traditionally, the “monoamine hypothesis” has dominated the understanding of this disorder, suggesting that depression is associated with a deficiency of monoamine neurotransmitters such as serotonin, norepinephrine, and dopamine (Cunningham et al., 2021; Pariante and Lightman, 2008).Recent research has expanded the scope of depression studies to include other mechanisms such as hypothalamic–pituitary–adrenal (HPA) axis dysregulation, inflammation, deficits in neuroplasticity, and alterations in glial cell function (Beurel et al., 2020; Mikulska et al., 2021; Price and Duman, 2020). Among these mechanisms, the role of astrocytes has garnered significant attention. Astrocytes are involved in neurotransmitter cycling, synaptic transmission regulation, and maintenance of the extracellular environment, all of which are critical for neuronal function and mood regulation (Zhao et al., 2022). Astrocyte are a type of glial cell in the brain that play essential roles in maintaining homeostasis, forming the blood–brain barrier, providing nutrients to the nervous tissue, and repairing the brain and spinal cord following traumatic injuries. In recent years, increasing research has highlighted the role of reactive astrogliosis in various neuropsychiatric disorders, including depression. This growing interest has spurred a substantial body of research and publications in the past decade (O’Leary and Mechawar, 2021).

Astrocytes interact with neurons and other glial cells, influencing brain function and behavior. They release various neurotrophic factors, cytokines, and chemokines that can affect neuronal survival, growth, and synaptic plasticity. In the context of depression, reactive astrogliosis are involved in several key processes. For example, they help regulate the levels of the primary excitatory neurotransmitter glutamate in the brain, preventing excitotoxicity, which can lead to neuronal damage and is associated with depressive symptoms (Onaolapo and Onaolapo, 2021). Additionally, reactive astrogliosis are involved in the brain’s inflammatory response, which has been linked to depression. Pro-inflammatory cytokines produced by astrocytes, such as interleukin-1*β* (IL-1β) and tumor necrosis factor-*α* (TNF-α), have been shown to induce depression-like behavior in animal models (De Tomaz et al., 2020). Reactive astrogliosis may also play a role in depression’s pathophysiology, opening up new therapeutic possibilities. Targeting astrocyte function and astrocyte-neuron interactions holds promise for more effective management of depression. For instance, drugs that modulate astrocyte activity, such as β-lactam antibiotics that enhance glutamate uptake, have shown potential in alleviating depressive symptoms in preclinical studies (Frizzo and Ohno, 2021). Moreover, astrocytes play a role in the mechanism of action of several antidepressant drugs. Selective serotonin reuptake inhibitors (SSRIs), commonly used antidepressants, have been found to increase the release of neurotrophic factors from astrocytes, which may contribute to their therapeutic effects (Chen et al., 2021).

Bibliometric analysis is a powerful tool that quantitatively assesses the scientific literature to evaluate the research landscape of a particular field (Diem and Wolter, 2013). It helps researchers identify trends, hotspots, and gaps in the literature, providing a comprehensive overview of the research domain (Mayr and Scharnhorst, 2015). In the context of astrocyte and depression research, bibliometric analysis can elucidate the development of this research area, highlight key contributions, and suggest future research directions (Merigó et al., 2015). The study aims to conduct a bibliometric analysis of research on the role of astrocytes in depression and its treatment from 2014 to 2023. Upon establishing our research topic, we conducted a literature search and found that studies exploring the association between astrocytes and depression date back to the 1990s. However, research in this area has significantly increased over the past decade. Therefore, to ensure that the analyzed data reflects current academic discussions and research directions, we have chosen this specific timeframe. By examining publication trends, citation patterns, and collaboration networks, we aim to provide a detailed map of the current research landscape in this field. This analysis will also identify the most influential studies, researchers, and institutions, thereby shedding light on the development and impact of research on astrocytes in depression. Ultimately, this work aims to guide future research efforts, foster collaboration, and deepen our understanding of the mechanisms and therapeutic potential of astrocytes in depression.

## 2 Methods

### 2.1 Data source and search strategies

**Figure 1:**
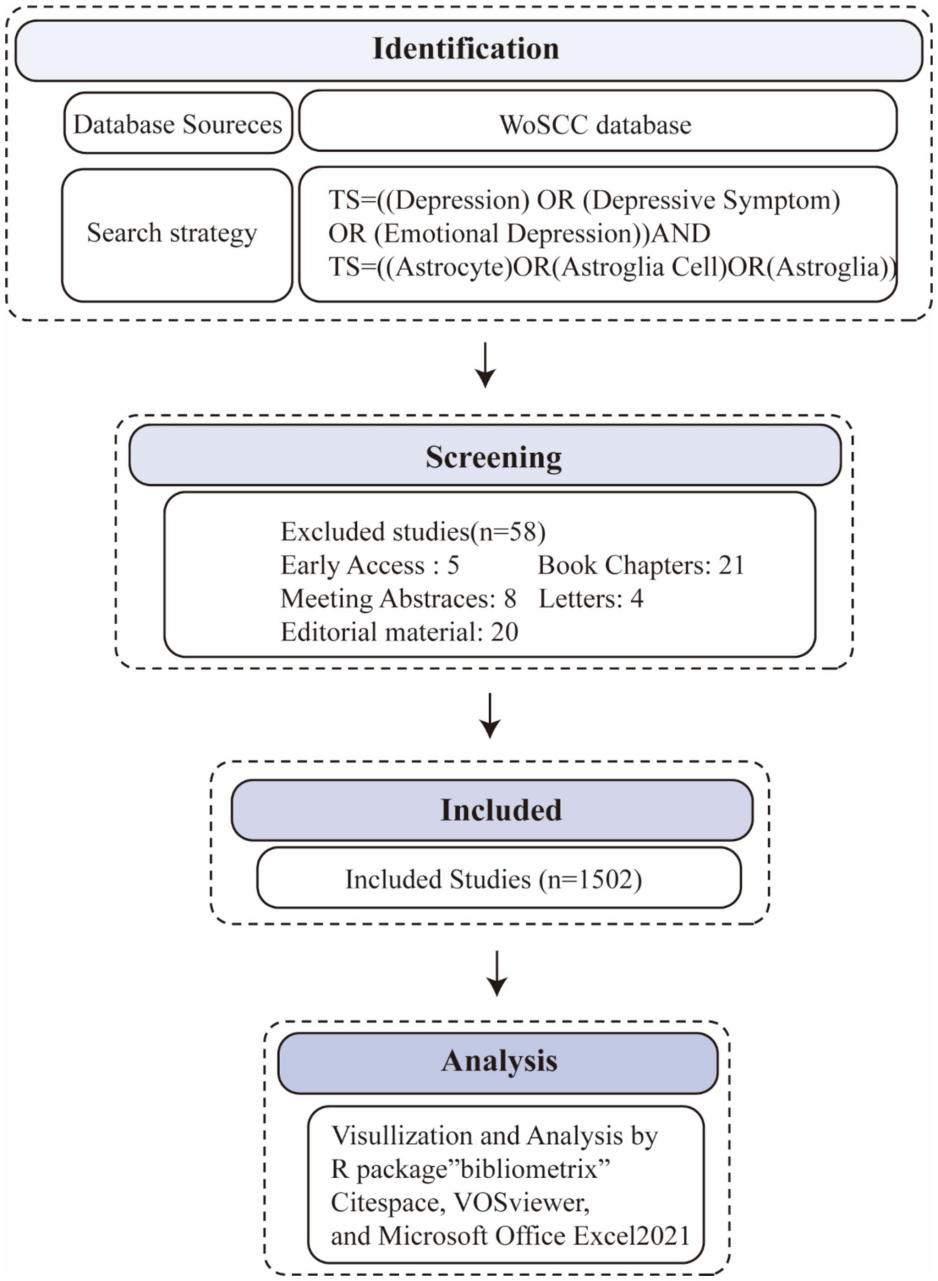
Flowchart of publication search and selection.

This study conducted a bibliometric analysis on the role of astrocytes in depression and its treatment, focusing on publications from 2014 to 2023. The Web of Science database was chosen as the primary data source due to its extensive coverage of high-impact scientific literature and was found to be the most suitable database for bibliometric analysis (Ding and Yang, 2022). The Web of Science (WoS) database was selected as the primary data source due to its peer-reviewed, high-quality research publications and its strong reputation within the academic community, making it one of the most suitable databases for bibliometric analysis. Other databases were excluded because of their overlapping coverage with WoS. Focusing on a single source ensured efficient data handling while providing a comprehensive view of the relevant literature, without the redundancy that could arise from using multiple similar databases. The Web of Science search formula was established as follows: TS = (Depressive Symptom* OR Symptom, Depressive OR Emotional Depression OR Depression) AND TS = (Astrocyte* OR Astroglia Cell* OR Astro glial Cell* OR Astroglia*). The inclusion criteria were as follows: (1) the literature type had to be original articles or reviews. The exclusion criteria included: (1) documents not published in English, and (2) editorial materials, retracted publications, book chapters, corrections, conference abstracts, conference papers, early access articles, letters, retractions, and publications expressing concerns. Two researchers (LLS and GZY) read the abstracts or full texts and screened them one by one. Discrepancies were resolved through discussion or consultation with a third researcher (RZY) (Figure 1).

### 2.2 Data analysis and visualization

Data analysis involved several bibliometric techniques (van Eck and Waltman, 2009), including VOSviewer (version 1.6.18), CiteSpace (version 6.1.R1), and the R package bibliometrix (version 3.2.1). Descriptive analysis was employed to outline the basic characteristics of the publications, including annual publication counts, journal distribution, and document types. Authorship and collaboration patterns were assessed using VOSviewer software to visualize co-authorship networks and identify leading researchers and institutions. Citation analysis measured the impact of articles, authors, and journals, employing metrics such as citation counts and the h-index. Keyword co-occurrence analysis was performed to identify frequently used terms and visualize research themes and hotspots through network maps created with VOSviewer (Donthu et al., 2021). Thematic analysis grouped articles into clusters based on keyword similarity, providing insights into main research topics and their evolution over time. Various visualization tools, including graphs, charts, and network diagrams, were employed to represent publication trends, collaboration networks, research hotspots, and the overall impact of research in this field (Ismail et al., 2024). This comprehensive methodological approach ensures a robust and insightful analysis of the scientific literature on astrocytes in depression, highlighting key trends, influential studies, and emerging research directions.

## 3 Results

### 3.1 Annual publications and trends

**Figure 2:**
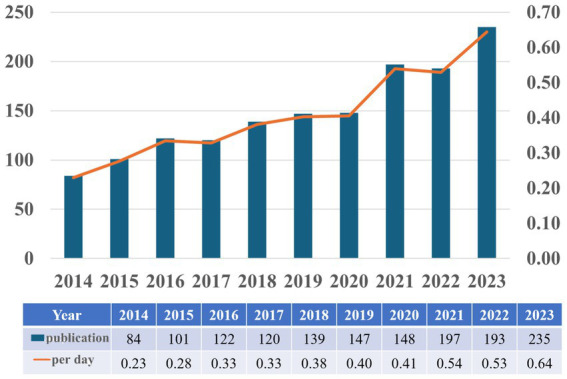
Annual scientific production on research into astrocytes role in depression from 2014 to 2023.

Based on our search strategy, the bibliometric analysis includes a total of 1,502 publications, comprising 1,213 research articles (80.76%) and 289 review articles (19.24%). Figure 2 illustrates the annual publication trends regarding astrocytes and depression from 2014 to 2023. Overall, there is an upward trend in the number of publications, with slight decreases of 2 publications in 2017 and 4 publications in 2022 compared to the previous years; however, these declines are not significant. The calculation of the average daily publication rate reveals an increase from an average of 0.23 publications per day in 2014 to 0.64 publications per day in 2023. This analysis indicates that interest in researching astrocytes and depression has been steadily growing over the past decade, and this upward trend may continue in the future.

### 3.2 Analyze the countries

**Figure 3:**
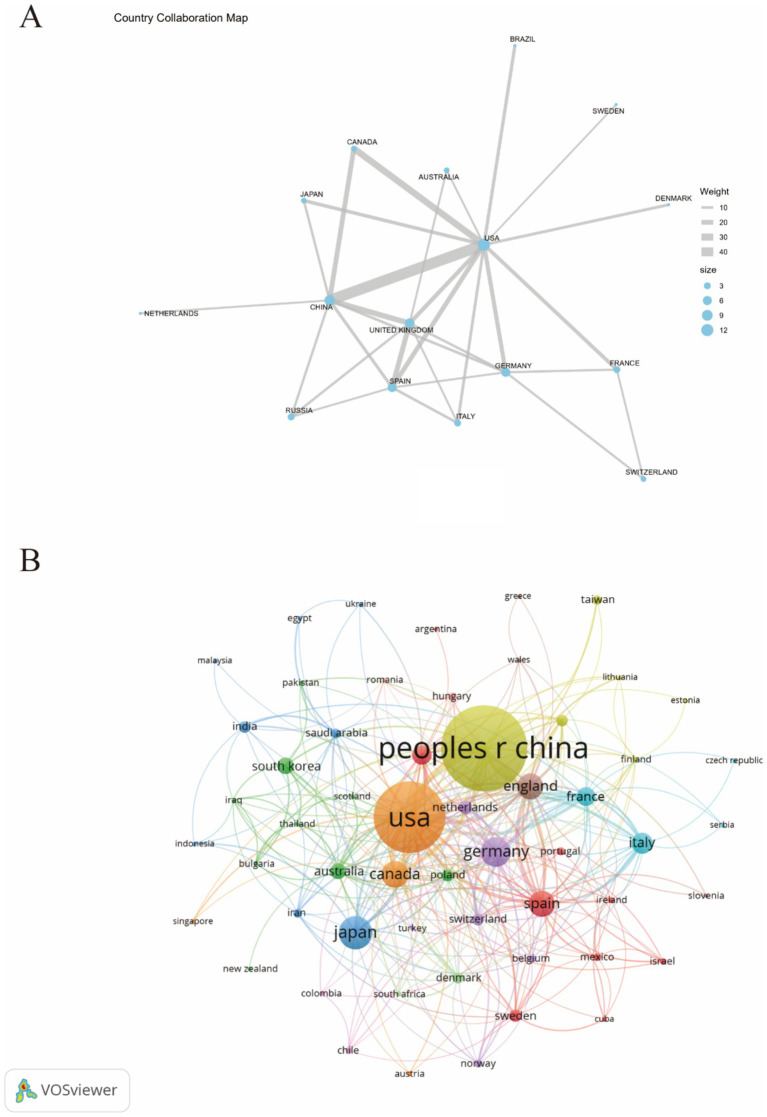
The publications density map of country **(A)** and visualization of countries **(B)** for research on astrocytes in depression.

Figure 3A illustrates the collaborative relationships between different countries through geographical positioning and connecting lines. The blue and brown shaded areas in the figure represent varying degrees of collaboration intensity among nations, providing a comprehensive view of the international cooperation network in this field. It is evident from the figure that China and the United States have the closest collaborative ties. Additionally, the United States also maintains strong cooperative relationships with countries such as Canada, the United Kingdom, and Germany, highlighting its broad global collaboration. Figure 3B further depicts the interrelationships and interactions between countries using a network diagram. In this diagram, the size of each node represents the significance of each country in collaboration, while the colors differentiate the various collaboration patterns between nations. Notably significant nodes include “USA” and “CHINA,” indicating that these two countries occupy a central position within the international cooperation network.

### 3.3 Analyze the international cooperation

**Figure 4:**
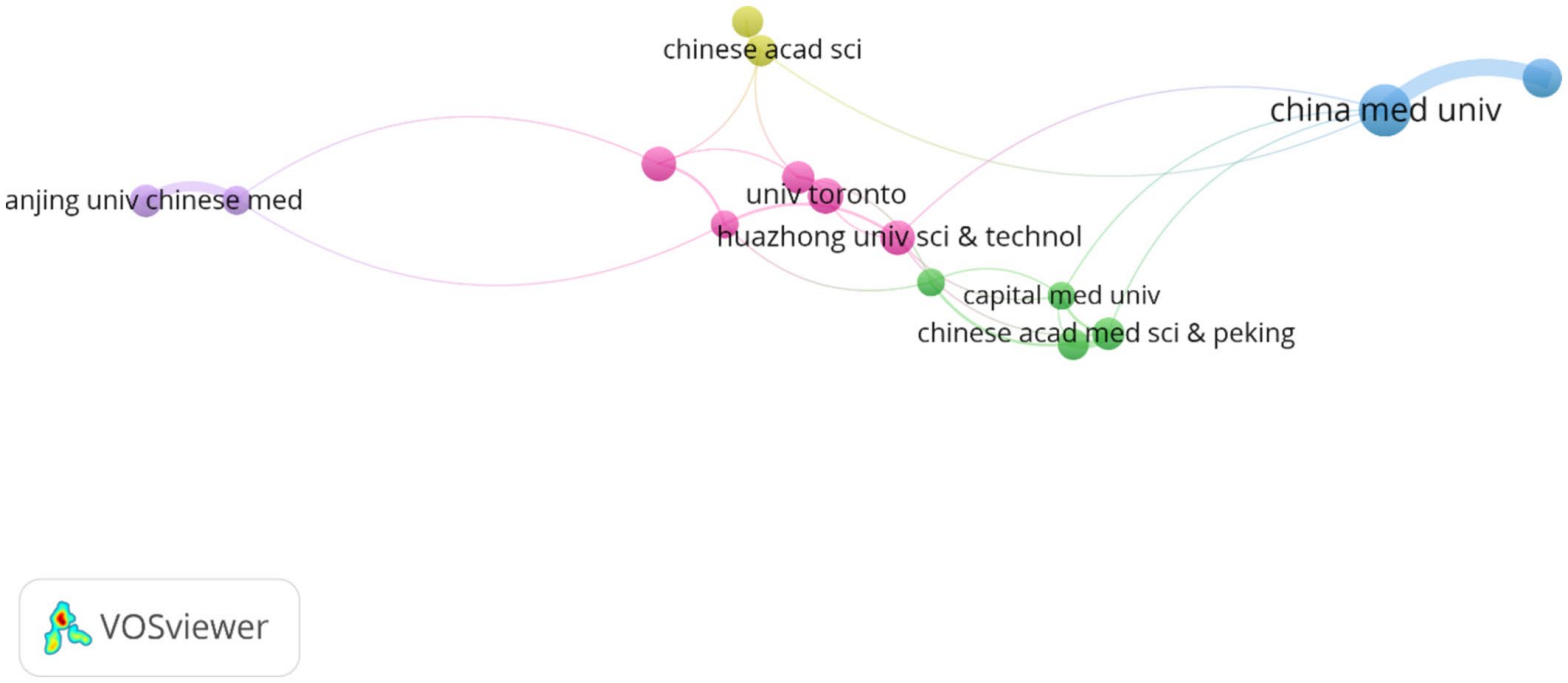
The visualization of institutions for research on astrocytes in depression.

The analysis of institutional contributions highlights that the top ten institutions are predominantly located in China, reflecting the country’s leading role in this research area. China Medical University was the most productive institution, with 32 publications (2.13%), followed by The University of Manchester in England with 24 publications (1.60%), and the University of Toronto in Canada with 22 publications (1.46%). Other key institutions included Huazhong University of Science and Technology (*n* = 21, 1.40%), Southern Medical University (*n* = 21, 1.40%), and the Chinese Academy of Medical Sciences and Peking Union Medical College (*n* = 20, 1.33%). McGill University in Canada, Nanjing University of Chinese Medicine, Beijing University of Chinese Medicine, and the University of Chinese Academy of Sciences also made significant contributions with 19 to 20 publications each, as detailed in Table 1. Figure 4 presents the collaborative network of global research institutions in this field. The different colored clusters in the figure indicate the distribution of various research directions and areas, highlighting the central position of Chinese institutions such as China Medical University, Huazhong University of Science and Technology, Nanjing University of Chinese Medicine, and Shandong University in this domain. Additionally, the figure illustrates the close collaborative relationships these institutions have with other research entities, including Karolinska Institutet in Sweden, Johns Hopkins University in the United States, and Universidade de São Paulo in Brazil.

**Table 1:** Top 10 countries and institutions on research of astrocytes in depression.

Rank	Country	Counts	Institution	Counts
1	China	489 (32.56%)	China Medical University (China)	32 (2.13%)
2	The United States	373 (24.83%)	The University of Manchester (English)	24 (1.60%)
3	Japan	122 (8.12%)	University of Toronto (Canada)	22 (1.46%)
4	Germany	104 (6.92%)	Huazhong University of Science and Technology (China)	21 (1.40%)
5	Canada	87 (5.79%)	Southern Medical University (China)	21 (1.40%)
6	England	86 (5.73%)	Chinese Academy of Medical Sciences and Peking Union Medical College (China)	20 (1.33%)
7	Spain	85 (5.66%)	McGill University (Canada)	20 (1.33%)
8	Italy	62 (4.13%)	Nanjing University of Chinese Medicine (China)	20 (1.33%)
9	Brazil	57 (3.79%)	Beijing University of Chinese Medicine (China)	19 (1.26%)
10	France	54 (3.60%)	University of Chinese Academy of Sciences (China)	19 (1.26%)

### 3.4 Analyze the journals and co-cited journals

**Figure 5:**
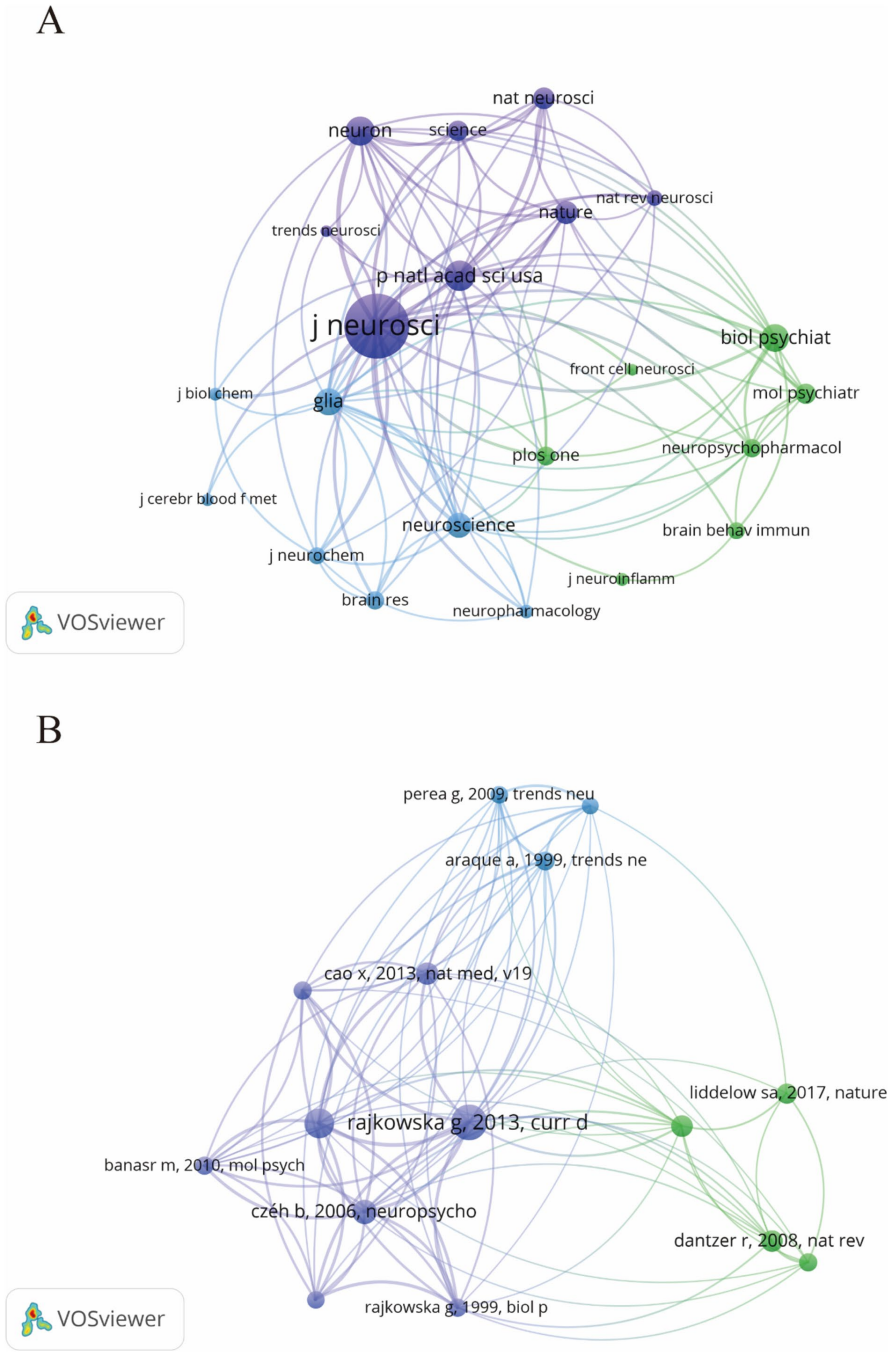
The visualization of journals **(A)** and co-cited journals **(B)** for research on astrocytes in depression.

From 2014 to 2023, research on astrocytes in depression and treatment was published across 169 journals. As shown in Table 2, among the top 15 co-cited journals, Frontiers in Cellular Neuroscience led with 47 articles (3.13%), followed by the International Journal of Molecular Sciences (43 articles, 2.86%) and Brain Behavior and Immunity (41 articles, 2.73%). High-impact journals included Brain Behavior and Immunity and Molecular Psychiatry. Figure 5 illustrates a citation network with journals as nodes. The purple section represents journals primarily focused on basic research, while the green section concentrates on biomedical and related applications. The most frequently cited journal is the Journal of Neuroscience, with 5,519 citations, followed by PNAS with 2,587 citations and Neuron with 2,515 citations. The co-cited journals exhibit significantly high impact factors, with Nature and Science being the journals with the highest impact factors in the co-citation group. They can be referred to as peer-reviewed journals, well-established journals, or high-impact journals. Figure 5B displays the key literature “Rajkowska and Stockmeier, 2013,” which shows multiple associated nodes with other literature. Different colors represent various research themes, while the density and thickness of the lines in the figure convey the strength of the citation relationships among these publications.

**Table 2:** Top 15 journals and co-cited journals for research on astrocytes in depression.

Rank	Journal	Counts	Journal	Co-citation
1	Frontiers in Cellular Neuroscience	47 (3.13%)	Journal of Neuroscience	5,519
2	International Journal of Molecular Sciences	43 (2.86%)	Proceedings of the National Academy of Sciences of the United States of America (PNAS)	2,587
3	Brain Behavior and Immunity	41 (2.73%)	Neuron	2,515
4	Journal of Neuroinflammation	32 (2.13%)	Biological Psychiatry	2,380
5	Glia	28 (1.86%)	Glia	2,303
6	Neuropharmacology	26 (1.73%)	Neuroscience	2,141
7	Scientific Reports	25 (1.66%)	Nature	1988
8	Frontiers in Pharmacology	23 (1.53%)	Nature Neuroscience	1835
9	Molecular Psychiatry	23 (1.53%)	Molecular Psychiatry	1735
10	Neuroscience	22 (1.46%)	Science	1731
11	Translational Psychiatry	22 (1.46%)	PLOS ONE	1,649
12	Behavioural Brain Research	21 (1.40%)	Neuropsychopharmacology	1,562
13	Frontiers in Neuroscience	20 (1.33%)	Brain Research	1,557
14	Molecular Neurobiology	19 (1.26%)	Brain, Behavior, and Immunity	1,483
15	Neurochemical Research	19 (1.26%)	Journal of Neurochemistry	1,477

### 3.5 Analyze the authors and co-cited authors

**Figure 6:**
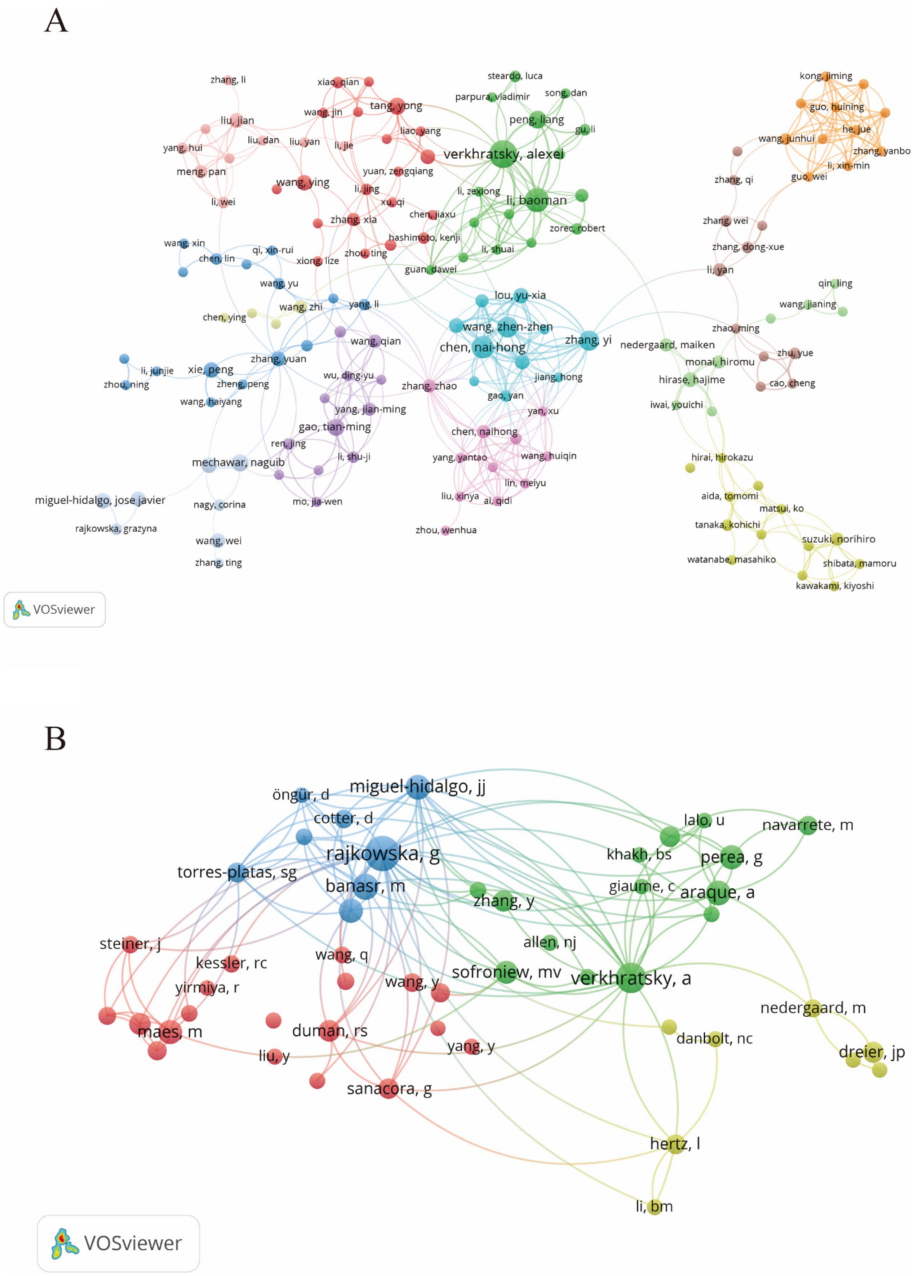
The visualization of authors **(A)** and co-cited authors **(B)** for research on astrocytes in depression.

During the study’s search period, a total of 8,759 authors contributed to research on astrocytes in depression and treatment. This study built a collaborative network based on authors who have published at least four papers. The figure illustrates the co-occurrence relationships among researchers in the literature. Nodes represent individual researchers, and edges signify the frequency of their co-appearance within the same documents. The size of each node indicates the significance of the researcher, as measured by their frequency of appearance in the literature. Moreover, nodes are color-coded based on clustering analysis results, categorizing them into different clusters that reflect thematic relationships or author groupings. Network visualization reveals each individual cluster, with Verkhratsky Alexei, Baoman Li, and Naihong Chen having the largest nodes because they have published the highest number of related works (Figure 6A). Among the 54,386 co-cited authors, the most co-cited author is Rajkowska Grazyna (*n* = 408). Notable among the top authors, Verkhratsky Alexei published 22 papers, followed by Baoman Li with 16 papers and Naihong Chen with 14 papers. Verkhratsky Alexei, with a co-citation count of 304, was also a highly influential figure in this field (Table 3). As shown in Figure 6B, the clustering of blue nodes represents academic communities with close collaboration in the research field of glial cells and their relationship with depression. Notably, prominent blue nodes include Verkhratsky Alexei and Baoman Li, who have made significant contributions to understanding the functions of glial cells and their roles in depression. They frequently collaborate on investigating the mechanisms of glial cells under various pathological conditions and exploring potential therapeutic strategies. In contrast, the green and red nodes represent researchers collaborating in other areas, such as neuroinflammation, the relationship between the gut microbiome and depression, neuroplasticity, and the interplay between metabolism and depression.

**Table 3:** Top 10 authors and co-cited authors for research on astrocytes in depression.

Rank	Authors	Counts	Co-cited authors	Citation
1	Verkhratsky Alexei	22	Rajkowska Grazyna	408
2	Baoman Li	16	Verkhratsky Alexei	304
3	Naihong Chen	14	Banasr Mounira	227
4	Zhenhen Wang	12	Araque Alfonso	207
5	Yi Zhang	11	Jose Javier Miguel-Hidalgo	201
6	Cai Song	10	Maes Michael	198
7	Gang Hu	9	Boldizsár Czéh	193
8	Liang Peng	9	Perea Gertrudis	191
9	Mechawar Naguib	8	Michael Victor Sofroniew	174
10	Yong Tang	8	Ronald S. Duman	157

### 3.6 Analyze the co-cited references

**Figure 7:**
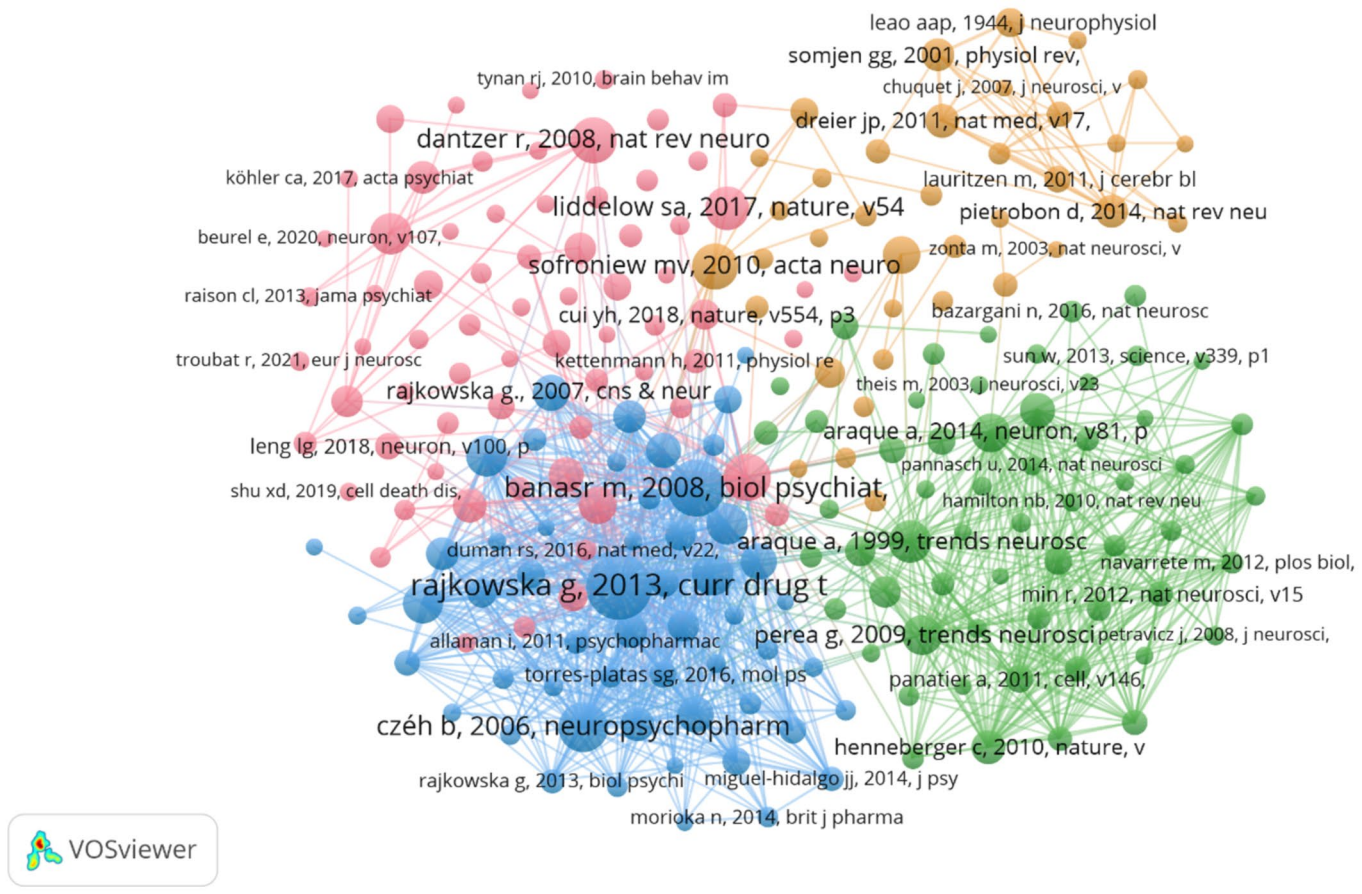
The visualization of co-cited references.

A total of 82,395 co-cited references were identified, indicating a robust interlinking of scholarly works in this domain. The co-citation network map (Figure 7) illustrates the interconnectedness of these influential studies. Nodes in the figure represent the cited literature, the size of nodes is proportional to the cited frequency of the literature, and the color and position are determined by cluster analysis and multidimensional scaling (MDS) algorithm. The network map is divided into three main color clusters that represent different areas of study or topics. The network is composed of several clusters, each representing different focal points within the broader research area. For instance, the red cluster includes works by Rajkowska and Dantzer, focusing on reactive astrogliosis pathology and the immune system’s influence on the brain, respectively. The green cluster, featuring studies by Araque, emphasizes the tripartite synapses and glial interactions. The cluster in blue mainly includes research on stress and stress response and the physiological and psychological effects associated with it, and this cluster literature focuses on the effects of stress on the brain and behavior, as well as its potential therapeutic pathways and mechanisms. Key references such as Sofroniew and Vinters (2010) on astrocyte biology and pathology, and Liddelow et al. (2017) on neurotoxic reactive astrocytes, further demonstrate the diversity and depth of research topics that coalesce around astrocytes in depression. The top 10 co-cited references (Table 4) serve as pivotal sources in understanding the role of astrocytes in depression. The most co-cited reference is Rajkowska and Stockmeier (2013), published in Current Drug Targets, with 140 co-citations. This study provides significant insights into reactive astrogliosis pathology in major depressive disorder, further demonstrating the diversity and depth of research topics that coalesce around astrocytes in depression.

**Table 4:** The top 10 co-cited references for research on astrocytes in depression.

Rank	Title	First author	Year	Journal	Co-citations
1	Astrocyte Pathology in Major Depressive Disorder: Insights from Human Postmortem Brain Tissue	Grazyna, Rajkowska	2013	Current Drug Targets	140
2	Glial Loss in the Prefrontal Cortex Is Sufficient to Induce Depressive-like Behaviors	Mounira, Banasr	2008	Biological Psychiatry	116
3	Astroglial Plasticity in the Hippocampus is Affected by Chronic Psychosocial Stress and Concomitant Fluoxetine Treatment	Boldizsár, Czéh	2006	Neuropsychopharmacology	96
4	Astrocyte-derived ATP modulates depressive-like behaviors	Xiong, Cao	2013	Nature Medicine	87
5	Astrocytes: biology and pathology	Sofroniew, Michael V	2010	Acta Neuropathologica	86
6	From inflammation to sickness and depression: when the immune system subjugates the brain	Dantzer, Robert	2008	Nature Reviews Neuroscience	84
7	Neurotoxic reactive astrocytes are induced by activated microglia	Liddelow, Liddelow	2017	Nature	80
8	Tripartite synapses: glia, the unacknowledged partner	Araque, Alfonso	1999	Trends in Neurosciences	75
9	Morphometric evidence for neuronal and glial prefrontal cell pathology in major depression	Grazyna, Rajkowska	1999	Biological Psychiatry	72
10	Gap Junction Dysfunction in the Prefrontal Cortex Induces Depressive-Like Behaviors in Rats	Jiandong, Sun	2012	Neuropsychopharmacology	72

### 3.7 Reference with citation bursts

**Figure 8:**
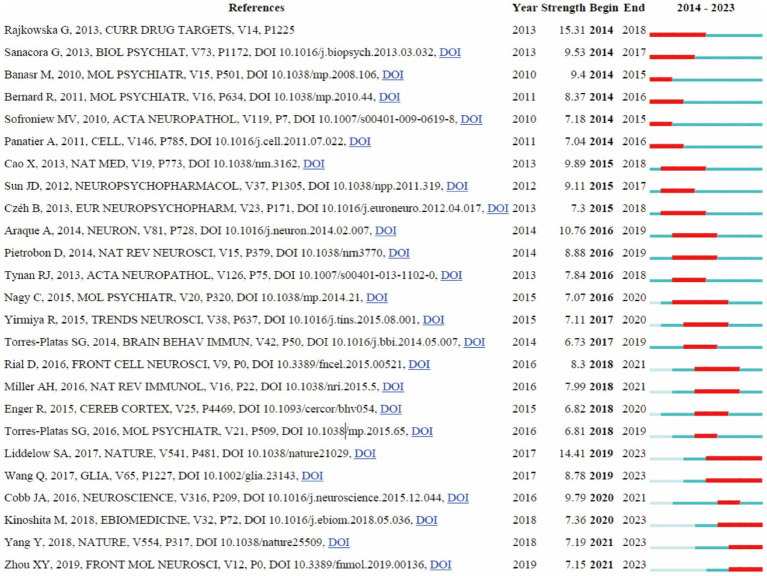
The visualization of citation bursts.

A detailed analysis of references with significant citation bursts was conducted to identify pivotal works that have substantially influenced research on astrocytes in depression (Amjad et al., 2022). A total of 25 references with the strongest citation bursts were identified by CiteSpace (Figure 8), emphasizing the substantial scholarly attention these works have garnered. The most prominent reference is Rajkowska and Stockmeier (2013) in “Curr Drug Targets,” which exhibited the highest citation burst strength of 15.31 from 2014 to 2018, underscoring its profound impact on the field. The second strongest citation burst (14.41), observed from 2019 to 2023, was attributed to Liddelow et al. (2017) in “Nature,” focusing on neurotoxic reactive astrocytes induced by activated microglia, highlighting evolving insights into astrocyte roles in neuroinflammation and depression. Other significant references include Smith (2014) in “Brain Behavior and Immunity” with a burst of 12.98 (2017–2021), Sofroniew and Vinters (2010) in “Mov Disorders” with a burst of 11.50 (2018–2022), and Cao et al. (2013) in “Mol Psychiatry” with a burst of 10.61. The burst strengths of these 25 references ranged from 6.73 to 15.31, and their endurance strengths spanned from 2 to 4 years, indicating sustained scholarly attention. This analysis reveals the enduring influence and critical milestones of these references on subsequent research, providing valuable insights into current and emerging trends in the study of astrocytes and depression.

### 3.8 Analyze the keywords

**Figure 9:**
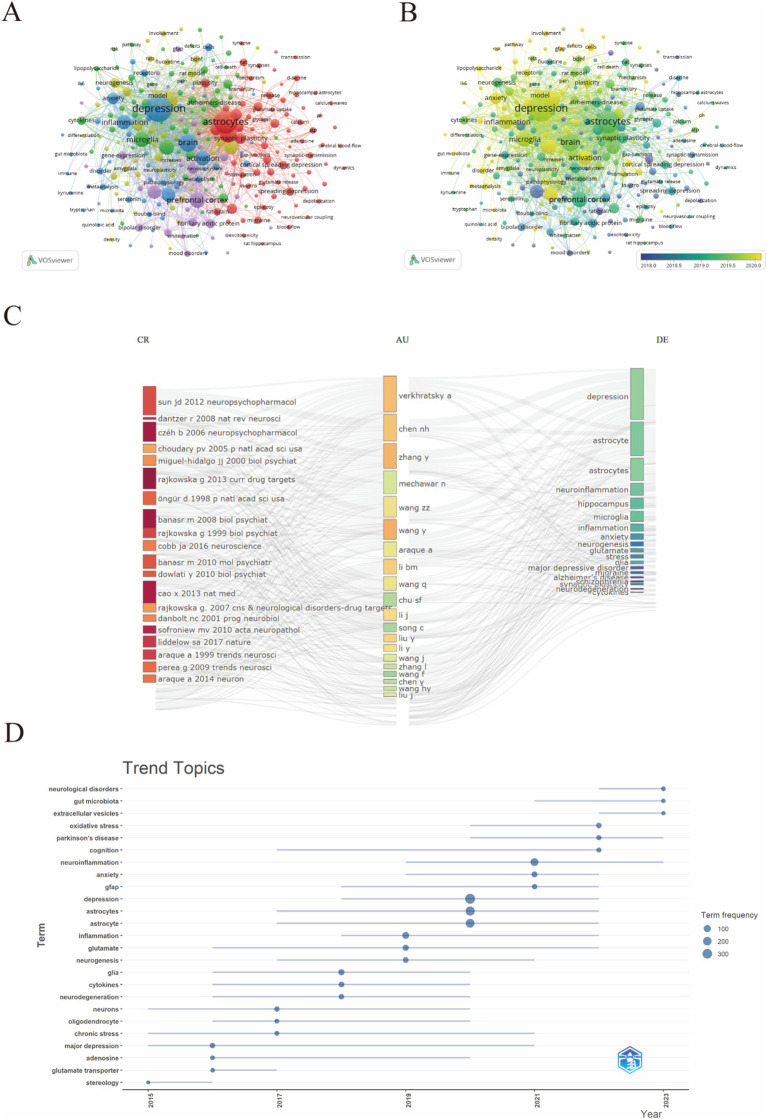
The visualization of keywords.

In this study, VOSviewer was used to create a keyword co-occurrence network view of 5300 documents, and 289 keywords with more than 10 occurrences were identified for visualization (Figure 9A), As shown in Figure 9A, the red cluster predominantly encompasses keywords such as “synaptic plasticity,” “astrocytes,” and “cortical spreading depression,” reflecting a concentration on the molecular and cellular mechanisms underlying depression. These terms are closely related to neuroscience and mental health. A greater number of connecting lines indicates a stronger relationship between two keywords. Similarly, the larger node for “astrocytes” indicates that this topic is widely discussed in the research. Key associated nodes include “microglia,” “neuron,” and “inflammation,” reflecting the close relationships between these terms and astrocytes. The blue cluster is primarily oriented toward the classification and treatment modalities of depression. Keywords within the purple cluster are mainly associated with the mechanisms of antidepressants. Additionally, the keyword distribution, based on the frequency of occurrence, was analyzed using VOSviewer. The overlay visualization map highlighted the average publication year for each keyword present in the articles, with the frequency of each keyword represented by the color intensity of the corresponding area. Before 2019, most studies concentrated on the themes of “prefrontal cortex” and “pathophysiology,” whereas the latest trends identified indicated that “inflammation” and “Microglia” might become future research emphases. (Figures 9A,B), revealing that keywords related to “astrocytes in depression” have gradually shifted from pathophysiological metabolism to inflammatory factors and intestinal microorganisms. The R Studio and bibliometric tools were used for analysis shown in Figure 9C, where the most used keywords are “depression,” “astrocytes,” and “neuroinflammation,” linked mainly to the most cited reference, which is the Sun JD (2012) Neuropsychopharmacology paper. The trend topic analysis, as depicted in Figure 9D, highlights evolving research priorities and emerging trends in the study of depression and astrocytes from 2015 to 2023. During this period, there is a notable increase in research on neurological disorders, gut microbiota, and Parkinson’s disease. Meanwhile, increased focus on inflammation, glutamate, and neurogenesis underscores the exploration of these mechanisms in depression.

## 4 Discussion

Bibliometrics serves as an effective tool for quantifying and analyzing the dynamic progression of specific research fields. Leveraging modern computational technology, it facilitates the visual representation of documents through clear and concise knowledge maps (Merigó et al., 2015). These maps enable researchers to gain a comprehensive understanding of the foundational aspects, current hotspots, and emerging trends within a field, transcending temporal and spatial limitations (Abramo et al., 2011). Consequently, bibliometrics is instrumental in aiding newcomers to swiftly acclimate to a particular research domain and enhance their document review efficiency. In this study, we performed a scientometric analysis to clarify current research trends on the role of astrocytes in depression treatment. Unlike systematic reviews, scientometric analyses provide the ability to predict future research directions within a specific field by synthesizing existing published findings (Fu et al., 2023).

**Figure 10:**
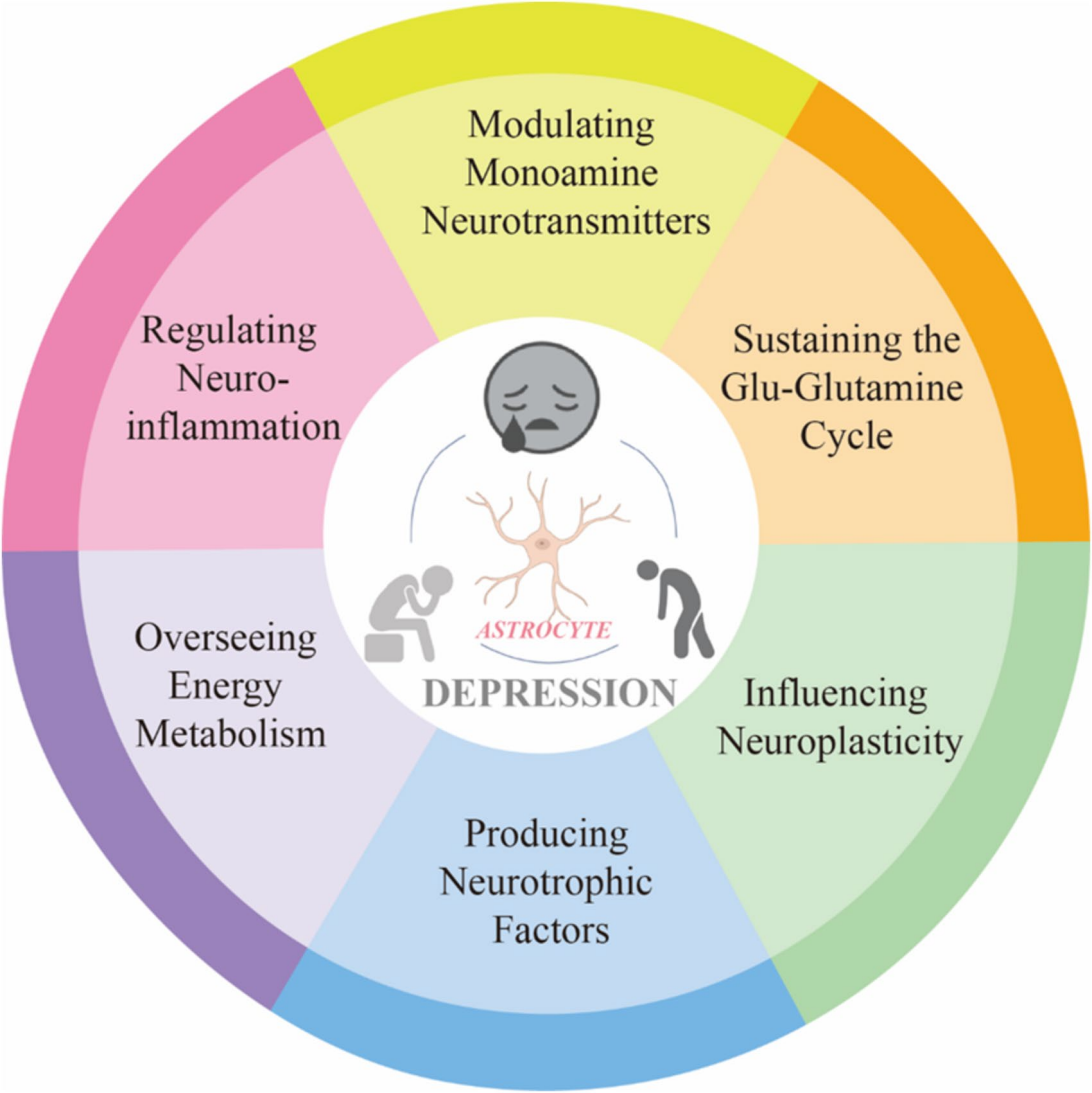
The relationship between astrocyte dysfunction and depression.

Depression is an emotional disorder characterized by persistent sadness and a lack of pleasure, with a global average prevalence of 4.4%. By 2030, depression is expected to become a leading cause of global disease burden and a major contributor to non-fatal health loss (Bayes.et al., 2020). Research has shown that astrocytes exhibit heightened sensitivity to stress and play a role in integrating peripheral and central stress responses (Lu. et al., 2024). Astrocyte, a type of glial cell within the brain, are the most abundant and widespread cells in the CNS. These cells exhibit complex structures and morphology, forming extensive astrocytic networks through gap junctions. They interact with neuronal synapses, blood vessels, and other glial cells, playing a significant role in the onset and progression of various neuropsychiatric disorders Astrocyte-mediated dysfunction appears to be involved in the pathophysiological processes of depression, making astrocytes an increasingly important focus in depression research (Zhao et al., 2022). This intricate relationship underscores the significance of astrocyte homeostasis for mental health. In our study, an analysis of annual publication trends over the past decade indicates a rising number of publications in this field. Specifically, the number of published articles increased from 84 in 2014 to 235 in 2023, reflecting a growing research interest in the complex interactions between astrocytes and depression. Given this trend, it is reasonable to speculate that this focus on astrocytes is likely to continue, thereby facilitating further research in this area. A bibliometric analysis of the literature from 2014 to 2023 reveals that prominent journals such as Frontiers in Cellular Neuroscience and International Journal of Molecular Sciences are leading in contributing influential research. Notably, China ranks first in research output, followed by the United States and Japan. However, it is worth noting that among the top ten research institutions, seven are based in China, indicating a robust research environment. Interestingly, there are no institutions from the United States or Japan in the top ten, which may suggest that research in these countries is more focused on the intersection of astrocytes and depression rather than being concentrated in a few major entities. The author collaboration network reflects the contributions of various institutions to this field. Visualization results show that Verkhratsky Alexei, Baoman Li, and Naihong Chen have the largest nodes, having published the most relevant works and demonstrating significant engagement in this area. With the emergence of new technological methods, these authors are likely to lead new frontiers in research. Interdisciplinary studies may inject fresh vitality into this field. A comprehensive analysis of co-cited references illustrates the research trends surrounding the complex relationship between astrocytes and depression. Works by Rajkowska and Dantzer focus on astrocyte pathology and the immune system’s effects on the brain, while Alarcon’s research emphasizes the interactions among tripartite synapses and glia. Key literature, such as Sofroniew and Vinters (2010) on astrocyte biology and pathology, and Liddelow et al. (2017) on neurotoxic reactive astrocytes, further demonstrates the diversity and depth of research themes surrounding astrocytes in depression. These references represent critical contributions to the ongoing discussions about astrocytes. Co-citation visualization analysis reflects the multidisciplinary nature of the research field. We utilized the VOSviewer analysis tool to study keyword co-occurrence and clustering, aiming to explore the forefront development trends in this area. Keywords such as “synaptic plasticity,” “neurons,” and “inflammation” indicate a concentrated investigation into the molecular and cellular mechanisms underlying depression. Overlay visualization maps highlight the average publication year of each keyword, revealing that prior to 2019, most research focused on themes of “prefrontal cortex” and “pathophysiology.” Recent trends suggest that “inflammation” and “microglia” may become future research priorities. This indicates a shift in the relevant keywords from pathophysiological metabolism to inflammatory factors and gut microbiota, suggesting a changing research paradigm. In-depth studies on the interactions between astrocytes and depression, as well as the development of appropriate prevention and treatment strategies, are becoming increasingly important. In summary, we analyzed the research trends related to the correlation between astrocytes and depression over the past decade. This provides guidance for researchers in the field. Future studies could expand the database scope and refine search strategies, thereby offering more precise insights into the relationship between astrocytes and depression (Figure 10).

### 4.1 Hotspots and Frontiers

#### 4.1.1 Keyword co-occurrence clustering analysis

Keywords capture the core essence of a document. Analyzing keyword co-occurrence can uncover research hotspots within a scientific field. The development trends of these hotspots can be more intuitively presented through a keyword co-occurrence network diagram over time (Chen, 2006). Emerging burst keywords can provide insights for future research based on the evolution of current research hotspots (Vanani and Jalali, 2017). The bibliometric analysis revealed several hotspots of research interest, such as ‘astrocytes’ and ‘neuroinflammation,’ identified through keyword co-occurrence and trend analyses. Recent years have seen an upsurge in studies exploring the gut-brain axis, with keywords such as ‘gut microbiota’ and ‘intestinal microorganisms’ appearing more frequently. This research frontier investigates how gut microbiota influence astrocytic function and contribute to the neurobiological underpinnings of depression, providing a new target for the treatment of depression by regulating the gut microbiome (Wang and Wang, 2016). On the other hand, advances in understanding astrocytic metabolism and its impact on brain function have shifted from basic pathophysiological metabolism to specific metabolic pathways involving inflammatory factors and energy homeostasis, highlighting how metabolic dysregulation in astrocytes can exacerbate depressive disorders (Anderson, 2022). Concurrently, the interplay between astrocytes and neurons, especially regarding depression, is a growing research area, with keywords like “astrocyte-neuron interactions” and “synaptic modulation” emphasizing efforts to elucidate complex signaling mechanisms between these cells, crucial for developing targeted therapies (Yao et al., 2023). However, current research on astrocyte mechanisms predominantly relies on rodent models, with relatively few clinical studies. Notably, behavioral tests in rodent models may not accurately predict antidepressant effects in humans, as these models do not fully capture the complexity of human depression (Chang and Hashimoto, 2024; Reardon, 2024).

In a word according to keyword clustering analysis and trend topic analysis, the most frequent keywords are “synaptic plasticity,” “astrocytes,” and “neuroinflammation,” indicating a focus on understanding the molecular and cellular mechanisms underlying depression. Recent studies have also explored the role of inflammatory factors and gut microbiota in astrocyte function and depression showcasing the evolving nature of this research area. Future research will likely focus on unraveling the intricate molecular and cellular mechanisms by which astrocytes contribute to depression.

#### 4.1.2 Research Frontiers of astrocyte in depression

##### 4.1.2.1 Monoamine neurotransmitters

The monoamine hypothesis of depression is a classical theory that posits a close relationship between the pathogenesis of depression and reduced levels of monoamine neurotransmitters, 5-HT, NE,and DA. Recent studies have indicated that astrocytes can significantly influence various serotonin receptors, including 5-HT1A, 5-HT2A, 5-HT2B, and 5-HT5A, as well as norepinephrine receptors such as α2A and β1B. Furthermore, the expression of both the serotonin transporter and the norepinephrine transporter has been observed in astrocytes (Peng et al., 2018). Interestingly, monoamine neurotransmitters have also been found to act on astrocytes, enhancing their metabolic processing of 5-HT and NE. Selective serotonin reuptake inhibitors (SSRIs) are classic antidepressants that exert their effects by inhibiting the activity of the serotonin transporter in astrocytes, leading to increased concentrations of 5-HT and ultimately manifesting antidepressant effects. Additionally, the 5-HT2B receptor has been confirmed as a potential target for classic antidepressants such as fluoxetine and sertraline, with abundant expression noted in astrocytes (Malynn et al., 2013). Research has shown that astrocytes in the striatum play a crucial role in maintaining dopamine homeostasis by assisting in the clearance of dopamine through various transport proteins during the dopamine transport process. This underscores the significant contribution of astrocytes to the regulation of dopaminergic signaling and highlights their importance in the overall neurochemical environment. By facilitating the reuptake and metabolism of dopamine, astrocytes not only support neuronal health but also contribute to the prevention of depression (Sočan et al., 2024). Thus, astrocytes play a dual role in the context of monoamine neurotransmission: they not only influence the secretion of these neurotransmitters but also are critical players in the process of neurotransmitter signaling. This highlights the importance of astrocytic involvement in the therapeutic mechanisms of antidepressants and underscores their potential as targets for novel treatment strategies in depression.

##### 4.1.2.2 Glu-glutamine cycle

The Excitatory Amino Acid Hypothesis posits that glutamate, a key neurotransmitter, and its associated excitatory signaling pathways play a critical role in the pathogenesis of mood disorders such as depression. Dysregulation of glutamate function may lead to the emergence of depressive symptoms (Narayan et al., 2022). The glutamate-glutamine cycle represents a vital metabolic pathway within the nervous system, involving both glutamate (the principal excitatory neurotransmitter) and glutamine (primarily synthesized and released by astrocytes) (Chan et al., 2020). In presynaptic neurons, glutamate is released into the synaptic cleft. Astrocytes are essential for the clearance and metabolism of glutamate, utilizing specific transporters such as GLT-1 and GLAST to reuptake glutamate from the synaptic cleft, thereby preventing excessive accumulation. This process is crucial for maintaining neuronal function and normal excitability. Once taken up by astrocytes, glutamate is converted into glutamine through the action of glutamine synthetase. Glutamine can then be transported back into neurons where it is reconverted into glutamate, thus sustaining neurotransmitter balance. Furthermore, astrocytes not only absorb glutamate but also release it, which can occur via a Ca^2+^-dependent mechanism and influence neuronal activity. The glutamate released by astrocytes can affect the excitability of neighboring neurons, thereby modulating synaptic transmission (Averill et al., 2020). When astrocytic function is impaired, the processing of glutamate and glutamine is affected, leading to a diminished ability for glutamate reuptake. This results in an accumulation of glutamate in the synaptic cleft, which may exacerbate depressive symptoms (Chen et al., 2019). Hence, astrocytes play a pivotal role in maintaining the stability of the nervous system through their involvement in the glutamate-glutamine cycle.

##### 4.1.2.3 Neuroplasticity

Changes in neural plasticity may be a key mechanism in the onset and progression of depression, as they affect the structure and function of neurons, thereby influencing emotional and cognitive abilities.The structure and function of synapses are influenced by their activity levels and undergo adaptive modifications, a phenomenon known as synaptic plasticity. This process is fundamental to brain function and is widely regarded as the cellular foundation of learning and memory (Kim and Diamond, 2002). Astrocytes play a crucial role in synaptic plasticity, with estimates suggesting that a single astrocyte can envelop more than 100,000 synapses. By extensively surrounding synapses and forming tripartite synapses with neurons, astrocytes can both respond to neuronal activity and regulate synaptic transmission (Schreiner et al., 2015). Thus, they are essential for the formation, maturation, and maintenance of synapses. Moreover, astrocyte-mediated phagocytosis is also critical in synaptic plasticity. Astrocytes can release various pro-synaptogenic molecules, such as ATP, D-serine, cholesterol, and TNF-*α*, which are involved in synapse formation, maturation, and signal transduction (Baldwin and Eroglu, 2017). Additionally, they can prune and eliminate synaptic structures through phagocytic receptors, thereby influencing synaptic plasticity. Astrocytes can also secrete cytokines that trigger complement cascade reactions, mediating the elimination of synapses by microglia, thus controlling synapse density (Vainchtein et al., 2018). D-serine, synthesized primarily by astrocytes, is a potent glycine receptor agonist that predominantly acts on NMDA receptors and plays a significant regulatory role in NMDA receptor-mediated synaptic plasticity, as well as in learning and memory. Studies have found that D-serine levels in the cerebrospinal fluid and blood of patients with depression are lower, suggesting that astrocyte dysfunction can impair D-serine synthesis, leading to reduced NMDA receptor activity and compromised synaptic plasticity, which may trigger depressive-like behaviors (Malkesman et al., 2012; Sultan et al., 2015). Therefore, impaired synaptic plasticity is one of the contributing factors to the onset of depression.

##### 4.1.2.4 Neurotrophic factors

The neurotrophic factor hypothesis posits that the deficiency of neurotrophic factors, such as BDNF, leads to a decline in neural plasticity, thereby playing a significant role in the onset and progression of depression (Phillips, 2017). Astrocytes, the most abundant glial cells in the central nervous system, are responsible for providing nutrients and supporting neuronal function. They have the capacity to synthesize and secrete neurotrophic factors, including BDNF, which promotes neuronal survival and plasticity. Consequently, astrocytes provide effective support for neurotrophic factors (Araki et al., 2021). By releasing these factors, astrocytes participate in the regulation of neural plasticity and repair mechanisms in the brain. In states of depression, the functionality of astrocytes may be compromised, leading to decreased levels of neurotrophic factors that negatively affect emotional and cognitive functions. This phenomenon indicates that astrocytes can modulate the expression of neurotrophic factors (Jing et al., 2023). Studies have demonstrated an association between astrocytic dysfunction and the development of depression; furthermore, enhancing the production and release of neurotrophic factors by astrocytes has been shown to help alleviate depressive symptoms.

##### 4.1.2.5 Energy metabolism

The energy metabolism hypothesis of depression posits a close relationship between depression and disruptions in cellular energy metabolism. This hypothesis emphasizes that mitochondrial function in patients is often impaired, resulting in insufficient energy production, which subsequently affects neuronal activity and neurotransmitter synthesis (Jiang et al., 2024). Additionally, abnormalities in glucose and lipid metabolism are commonly associated with depression, and these metabolic disturbances may further exacerbate declines in emotional and cognitive functioning. Chronic inflammation also interferes with energy metabolism, creating a vicious cycle. Therefore, improving energy metabolism may represent a novel strategy for treating depression (Milaneschi et al., 2019). Astrocytes play a crucial role in energy metabolism by taking up and metabolizing glucose to provide the necessary energy for neurons, particularly through the conversion of glucose into lactate for neuronal utilization. Furthermore, astrocytes regulate neurotransmitter levels and maintain ionic concentration and pH balance within the brain, thereby supporting normal neuronal function. In response to metabolic stress and inflammation, astrocytes can adjust their metabolic activities to protect neurons. Consequently, astrocytes contribute significantly to the interplay between energy metabolism and the pathogenesis of depression through multiple mechanisms (Zhang et al., 2023). Firstly, they are responsible for the uptake and metabolism of glucose, converting it to lactate to supply essential energy to neurons; inadequate energy supply may impair emotional and cognitive functions, increasing the risk of depression. Secondly, astrocytes modulate levels of neurotransmitters, such as glutamate and GABA, maintaining homeostasis in the neural environment and protecting neurons from toxicity due to excessive neurotransmitter levels. Additionally, astrocytes play a pivotal role in responding to inflammatory processes in the brain. Chronic low-grade inflammation may disrupt their normal functions, contributing to a vicious cycle that exacerbates depressive symptoms. They also maintain ionic equilibrium to ensure normal neuronal excitability; disruptions in ionic homeostasis may lead to mood disorders (Marina et al., 2016). Finally, inadequate metabolic adaptability of astrocytes in response to external stressors may further lead to dysregulated energy metabolism. Thus, dysfunction of astrocytes is closely related to depression and represents a significant factor in its pathogenesis.

##### 4.1.2.6 Neuroinflammation

Excessive neuroinflammation is a hallmark of neurodegenerative diseases. Reactive astrogliosis, due to their reactive nature, are prone to significant changes in response to various pathological conditions, which can contribute to neuroinflammation and neurodegeneration (Neal and Richardson, 2018). Increasing evidence indicates that the excessive activation of neuroinflammation within the brain is primarily driven by the interaction between astrocytes and microglia, leading to an imbalance of inflammatory factors, this imbalance is one of the important mechanisms that contribute to the development of depression (Neal and Richardson, 2018). Activated glial cells in the neuroinflammatory environment affect neuronal injury and death through the production of glutamate, tumor necrosis factor *α* (TNF-α), prostaglandin, reactive oxygen species and active nitrogen and other neurotoxic factors, which interact with each other to further aggravate neuroinflammation (Niranjan, 2014). It has shown that the expression of pro-inflammatory factors is significantly elevated in the serum and cerebrospinal fluid of patients with depression (Liddelow et al., 2017). Additionally, individuals with inflammatory diseases or those receiving pro-inflammatory cytokine interferon-α therapy are more susceptible to depression compared to healthy individuals (Machado et al., 2017). Moreover, studies in animal models have demonstrated that pro-inflammatory factors can induce depressive-like behaviors (Zhang et al., 2015), which are negatively correlated with the levels of BDNF gene expression and protein (Lu et al., 2021). In pharmacological treatment, breviscapine, a traditional Chinese medicine monomer with antidepressant effects, can alleviate neuroinflammation-induced depressive-like behavior by inhibiting the activation of astrocytes (Lu et al., 2021). Treatment with fluorocitrate, an astrocyte metabolism inhibitor, improves depressive-like behavior by reducing the release of central pro-inflammatory factors (Zhang et al., 2020). Additionally, clinical antidepressants, such as sertraline and paroxetine, exert therapeutic effects on depression by inhibiting the excessive activation of the inflammatory system (Taler et al., 2007). In summary, neuroinflammatory responses induced by the release of inflammatory factors from reactive astrogliosis may be one of the key mechanisms underlying the development of depression. Therefore, targeting the inhibition of reactive astrogliosis to reduce neuroinflammatory responses could represent a novel therapeutic strategy for depression.

In summary, through the analysis of scientific literature, as research into the pathogenesis and therapeutic targets of depression deepens, the focus of development has gradually shifted from neurons to astrocytes. The primary areas of interest include pathological changes such as synaptic plasticity, astrocyte number and morphology, and neuroinflammation. The primary areas of interest include pathological changes such as synaptic plasticity, astrocyte number and morphology, and neuroinflammation (Figure 10). Therefore, exploring the mechanisms and therapeutic potential of astrocytes in depression is of significant importance.

## 5 Conclusion

As the first bibliometric analysis provides a detailed overview of research trends in astrocytes’ role in depression from 2014 to 2023. The rapidly increasing number of publications indicates that research on astrocytes in depression is gaining significant attention from scholars worldwide, with China and the United States leading the field. Key findings highlight astrocytes’ critical functions, emphasizing molecular mechanisms like synaptic plasticity, neurotransmission, and neuroinflammation. Emerging frontiers include the gut-brain axis, astrocytic metabolism, and astrocyte-neuron interactions, presenting new therapeutic potentials. The study underscores the necessity for integrated approaches in research and treatment development. Future directions should focus on these multifaceted roles to advance understanding and create effective astrocyte-targeted therapies for depression.

## 6 Strength and limitations

The strength of this bibliometric analysis lies in its comprehensive evaluation of the literature on the role of astrocytes in depression from 2014 to 2023. Utilizing tools like VOSviewer and CiteSpace, we effectively visualized and analyzed a substantial dataset, providing valuable insights into research trends, hotspots, and influential works. However, the study is not without limitations. The reliance on the Web of Science Core Collection as the primary data source may exclude relevant studies from other databases, impacting the comprehensiveness of the analysis. Furthermore, bibliometric analyses tend to favor more recent and frequently cited publications, potentially overlooking seminal works. Lastly, the focus on co-citation networks may not fully capture the contributions of all influential authors in the field (DeVito and Goldacre, 2019). Future research should consider integrating multiple databases to enhance data inclusivity and accuracy. Additionally, expanding the scope to include clinical studies will provide a more holistic view of the field and foster the translation of basic research findings into clinical practice.

## Data Availability

The original contributions presented in the study are included in the article/supplementary material, further inquiries can be directed to the corresponding author/s.
